# Atypical El Tor *Vibrio cholerae* from the second major global seventh-pandemic cholera wave is endemic in Sabah, Malaysia

**DOI:** 10.1128/spectrum.02191-25

**Published:** 2026-02-09

**Authors:** Jaeyres Jani, Jecelyn Leaslie John, Lia Natasha Amit, Deborah Yebon Kang, Marilyn Charlene Montini Maluda, Mohammad Jikal, Yann Felix Boucher, Kamruddin Ahmed

**Affiliations:** 1 Department of Biomedical Sciences, Faculty of Medicine and Health Sciences, Universiti Malaysia Sabah 106089 , Kota Kinabalu, Sabah, Malaysia; 2 Borneo Medical and Health Research Centre, Faculty of Medicine and Health Sciences, Universiti of Malaysia Sabah 106089 , Kota Kinabalu, Sabah, Malaysia; 3 Department of Pathology and Microbiology, Faculty of Medicine and Health Sciences, Universiti of Malaysia Sabah 106089 , Kota Kinabalu, Sabah, Malaysia; 4 Singapore Centre for Environmental Life Sciences Engineering (SCELSE), National University of Singapore 37580, Singapore, Singapore; 5 Sabah State Health Department, Ministry of Health Malaysia 26691 , Putrajaya, Malaysia; 6 Saw Swee Hock School of Public Health, National University Singapore and National University Health System, Singapore, Singapore; 7 Infectious Diseases Translational Research Program, Department of Microbiology and Immunology, Yong Loo Lin School of Medicine, National University of Singapore and National University Health System, Singapore, Singapore; University of Valencia, Valencia, Spain, USA

**Keywords:** *Vibrio cholerae*, whole-genome sequencing, atypical El Tor strains, CTX prophage, cholera epidemiology

## Abstract

Cholera, caused by *Vibrio cholerae*, remains a significant diarrheal disease, especially in coastal regions of developing countries. In Malaysia, cholera is largely non-endemic except in Sabah, which has had recurrent outbreaks accounting for ∼75% of national cases between 2004 and 2014. To understand the origin and transmission of the disease, we sequenced the genomes of clinical isolates of *V. cholerae* O1 collected during an outbreak in 2019 and 2020. Genotypic analyses revealed that all Sabah isolates were atypical El Tor biotype harboring Classical CTX prophage elements. In particular, the strains carried two tandem CTX prophage copies in chromosome 2 and three tandem RS1 sequences on chromosome 1, including a Classical type *rstR*, which is atypical for canonical El Tor. Genome comparisons revealed conserved seventh-pandemic genomic islands (VSP1 and VSP2) and variably arranged biotype-specific loci, suggesting pandemic-lineage markers and mobile elements linked to environmental adaptation. Phylogenetic reconstruction placed the Sabah strains within wave 2 of the seventh-pandemic clade, forming a distinct subclade with two genotypes, consistent with regional endemicity over the last few decades. Although wave 3 strains have largely replaced wave 2 globally, an established population of wave 2 strains in Southeast Asia suggests that they are more resilient than previously thought.

**IMPORTANCE:**

This study addresses a critical public health concern by investigating the genomic characteristics of *Vibrio cholerae* O1 strains responsible for recurrent cholera outbreaks in Sabah, Malaysia. Although cholera is largely non-endemic in most parts of Malaysia, Sabah remains an exception, contributing disproportionately to national case counts. By sequencing clinical isolations from the 2019 and 2020 outbreaks, this research provides essential insights into the origins, evolutionary dynamics, and transmission patterns of *V. cholerae* in a region with persistent endemicity. These findings underscore the importance of continuous genomic surveillance in geographically distinct settings and offer valuable data for informing public health strategies aimed at cholera control and prevention in Southeast Asia.

## INTRODUCTION

*Vibrio cholerae*, the etiologic agent of cholera, remains a significant cause of bacterial diarrhea in developing countries, especially coastal areas. Among more than 200 *V. cholerae* serogroups that have been discovered, two serogroups, O1 and O139, are mainly linked with major epidemics (1). Serogroup O1 is further categorized into two biotypes, the Classical and El Tor. The Classical biotype was behind the first six pandemics between 1899 and 1923 (2, 3), while the El Tor biotype is known to have caused the seventh and ongoing pandemic, which was first reported in Indonesia in 1961. Serogroup O139 was identified in the Indian subcontinent in 1992 and then became widespread in Southeast Asia (2–4). *V. cholerae* notably has two circular chromosomes, with chromosome 1 carrying essential housekeeping genes and chromosome 2 containing genes involved in environmental adaptation, stress response, and pathogenicity (5).

The El Tor biotype, first identified in 1905 at Egypt’s El Tor quarantine station (6), became epidemic in Indonesia before spreading across Asia, Africa, the Middle East, parts of Europe, Latin America, and lately Hispaniola in 2010 (3). Globally, cholera affects 1.3–4 million people annually, resulting in an estimated over 95,000 deaths (7). Previous studies have performed high-resolution single-nucleotide polymorphism (SNP) analyses of whole genomes, but these analyses represent only isolates from Peninsular Malaysia, which is approximately 640 km away from Sabah. In Malaysia, cholera has been documented since the 1950s, but it is now considered non-endemic, except in Sabah, which is located in East Malaysia (8). Outbreaks in Sabah, predominantly caused by the El Tor O1 serogroup, have been linked to contaminated food and water. Between 2004 and 2014, 3,481 cases and 32 deaths were reported nationwide, with Sabah accounting for 75.4% of the cases in Malaysia. Cholera outbreaks in Sabah peak every 3–5 years, with the highest incidence occurring along the east coast. In 2018, 163 cases were recorded across nine districts, with Semporna having the highest rate (51.5 per 100,000) (9). Given Sabah’s significant cholera burden, further research is crucial to identify risk factors and improve control measures. A detailed investigation into a 2014 cholera outbreak among Sea Gypsies (Bajau) in Kudat, with 44 symptomatic and 34 asymptomatic cases (10), revealed the main cause to be *V. cholerae* El Tor of the O1 Ogawa serotype. A retrospective study of strains isolated in 2015 across Sabah identified genetically diverse atypical El Tor strains among 65 clinical isolates and one environmental isolate through DNA fingerprinting (11). However, their origin and exact genotypes have remained a mystery, since whole-genome sequencing could not be performed at the time.

In May 2019, an outbreak of cholera occurred in the Putatan district of Sabah, and seven isolates could be obtained from patients. The following year, additional cases were reported across the state, and another 18 strains were isolated from clinical cases at local hospitals. Genome sequencing, followed by biotyping and phylogenetic analysis, revealed that the outbreak was caused by atypical *V. cholerae* El Tor O1 from the second wave of the current seventh pandemic.

## MATERIALS AND METHODS

### Strains and genome DNA preparation

**Fig 1:**
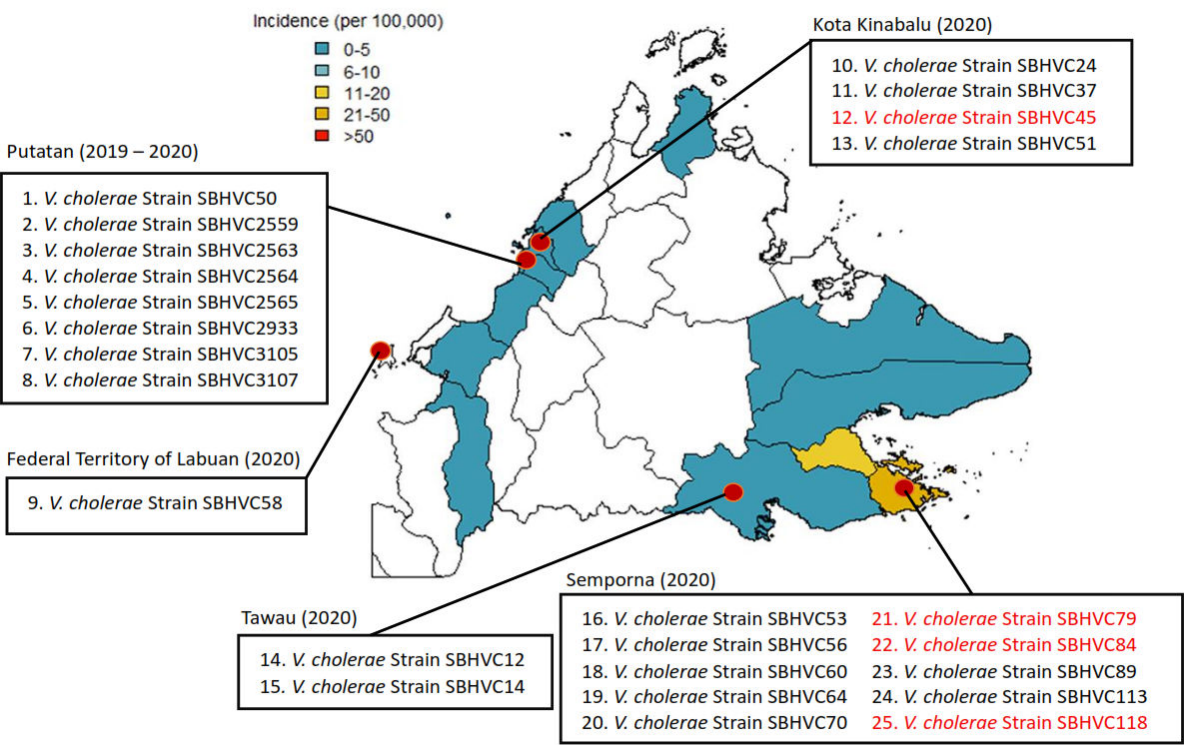
Map of Sabah showing the locations of isolated *V. cholerae* strains, including Kota Kinabalu, Putatan, Tawau, Semporna districts, and the Federal Territory of Labuan. The red-colored strains belong to a specific monophyletic subclade of wave 2, including three strains from Semporna and one from Kota Kinabalu. The incidence rate per 100,000 is shown on the map for each district of Sabah, Malaysia, for the year 2020 (9).

Twenty-five *V. cholerae* strains were isolated from stool samples collected from patients with cholera-induced diarrhea across different districts in Sabah (Fig. 1; Table S1). The initial diagnosis involved culturing the stool samples on Thiosulfate-Citrate-Bile-Salts-Sucrose agar. Genomic DNA from the 25 *V. cholerae* strains was extracted from overnight cultures grown at 37°C using a genomic DNA purification kit (QIAGEN, Hilden, Germany), following the manufacturer’s instructions. The quality and quantity of the extracted DNA were assessed using a Nanodrop spectrophotometer.

### Illumina and SMRTbell library preparation and sequencing

Strains of 25 *V. cholerae* were sequenced using the Illumina Novaseq 6000 system (Illumina, San Diego, CA, USA), generating 150 bp paired-end (PE) reads. All sequencing operations followed the protocols and reagents described in the manufacturing protocol. The genomic DNA library was prepared with the Nextera XT DNA Sample Prep Kit (Illumina). Briefly, 1 ng of input DNA was tagmented by the Nextera XT transposome at 55°C for 5 min, followed by end-repair, A-tailing, adaptor ligation, and library amplification, according to the manufacturer’s protocol. The DNA library was validated using the Agilent Bioanalyzer (Agilent Technologies, Santa Clara, CA, USA) and the Qubit Fluorometer (Thermo Fisher Scientific) for quality control analysis. The library was then denatured, diluted to the optimal concentration, and used for cluster generation with the NovaSeq 6000 S1 Reagent Kit (Illumina) on the flow cell. Image analysis and base calling were performed using SCS2.8/RTA1.8 (Illumina). FASTQ file generation and the removal of failed reads were conducted using CASAVA ver.1.8.2 (Illumina).

*V. cholerae* strain SBHVC14 was also sequenced by Macrogen Inc. (Geumcheon-gu, Seoul, South Korea) using a PacBio RS II sequencer with one SMRT cell and P6-C4 chemistry at a 120-min movie length (Pacific Biosciences, Menlo Park, CA, USA). A 20-kb SMRTbell library was generated from sheared genomic DNA using a 20-kb template library preparation workflow, following the protocols and reagents provided by the manufacturer.

### Hybrid genome assembly of *V. cholerae* strain SBHVC14

The genome of *V. cholerae* strain SBHVC14 was successfully assembled using a hybrid sequencing and assembly methodology. Long-read data generated from the PacBio RS II platform (P6-C4 chemistry) were first assembled using Canu, producing 16 contigs with a total length of 4,345,299 bp, an *N*_50_ of 1,053,043 bp, and a longest contig of 2,024,187 bp. While this initial assembly provided a comprehensive structural overview of the genome, it was further refined through error correction using high-accuracy Illumina short reads. These reads were aligned to the Canu V2.2 assembly contig using BWA-MEM2 V2.21, and the resulting alignments were polished with Pilon V1.24. This polishing process corrected sequencing errors and resolved local misassemblies, resulting in a significantly improved assembly with only two large scaffolds, with a total length of 4,293,028 bp (Table S2). The near-complete final assembly, with a consistent GC content of ~47.45%, demonstrates the effectiveness of integrating long-read and short-read sequencing platforms. Overall, this hybrid method not only enhanced genome continuity and accuracy but also provided a high-quality reference for downstream genomic and comparative analyses.

### Genome mapping to *V. cholerae* strain SBHVC14

The reads of 24 *V. cholerae* strains sequenced using Illumina technology were first cleaned using trim BBDuk (bbduk.sh) version 38.90 to remove adapter sequences and trim reads with quality scores lower than Phred score Q20. Next, the cleaned reads were mapped to each chromosome of the *V. cholerae* strain SBHVC14 using BWA v0.7.13. A consensus sequence was then generated from the mapping results (Table S2).

### Genome annotation and bioinformatics analyses

The genome annotation for each assembly was performed using the Prokka pipeline, which enables rapid and accurate annotation of bacterial genomes. Prodigal v3.3.6 was used to identify coding sequences, transfer RNAs (tRNAs), ribosomal RNAs (rRNAs), and other genomic features. tRNAs were predicted using tRNAscan-SE v2.0 (12), a specialized tool for detecting tRNA genes with high sensitivity and accuracy. 5S, 16S, and 23S rRNAs were identified using barrnap v0.9, which uses hidden Markov models. The presence of potential virulence factors and antibiotic resistance genes was identified using CholeraFinder v1.0. CheckM v1.1.6 was used to estimate genome completeness and contamination levels based on lineage-specific marker genes.

### Phylogenetic analysis and genome visualization

For comparative genomic analysis, SNPs were identified using the kSNP3 v3.1.2 tool. To accurately position the samples within a phylogenetic framework, the analysis included 62 previously published *V. cholerae* genomes retrieved from the NCBI database, as detailed in Table S3. A maximum likelihood phylogenetic tree was constructed using MEGA X based on the SNP-derived distance matrix, applying the general time reversible (GTR) model with 1,000 bootstrap replicates to assess the robustness of the inferred relationships. For genome-wide visualization, Circos (13) was used to generate circular genomic plots of 25 Sabah strains together with reference strains, providing an integrated view of the comparative genomic landscape.

## RESULTS AND DISCUSSION

### The 2019–2020 Sabah cholera outbreak occurred primarily in coastal areas but affected a geographically and demographically diverse population

The *V. cholerae* isolates collected during the 2019–2020 outbreak were obtained from patients residing in various districts across Sabah, including Semporna, Putatan, Kota Kinabalu, Tawau, and the Federal Territory of Labuan (Fig. 1). Among these, Semporna was the most affected district, with an incidence rate of 6.5 and 31.2 per 100,000 population in 2019 and 2020, respectively. This was followed closely by Putatan, with incidence rates of 11.4 and 4.3 per 100,000 population in those respective years. This geographic distribution suggests the presence of localized clusters of infection, possibly indicating environmental or community-based sources of transmission (9). In terms of gender distribution, approximately two-thirds of the total cases were female patients, whereas male patients comprised the remaining. This skew may reflect differences in exposure risks, health-seeking behavior, or report patterns between genders in the affected communities. The age of the patients varied widely, ranging from 4 to 83 years old, and a large proportion was young to middle-aged adults. This indicates that the outbreak affected a broad age range, including both pediatric and elderly populations (Table S1). Some patient records lacked complete demographic information, such as age or gender, which may limit demographic interpretation of the analysis. Nevertheless, the available data clearly depict a diverse demographic impact of cholera infections, affecting individuals across a broad spectrum of ages and both sexes, and concentrated in specific coastal and semi-urban districts of Sabah.

### Comparative genomics reveals atypical hybrid El Tor O1 characteristics in Sabah *V. cholera*e

**Fig 2:**
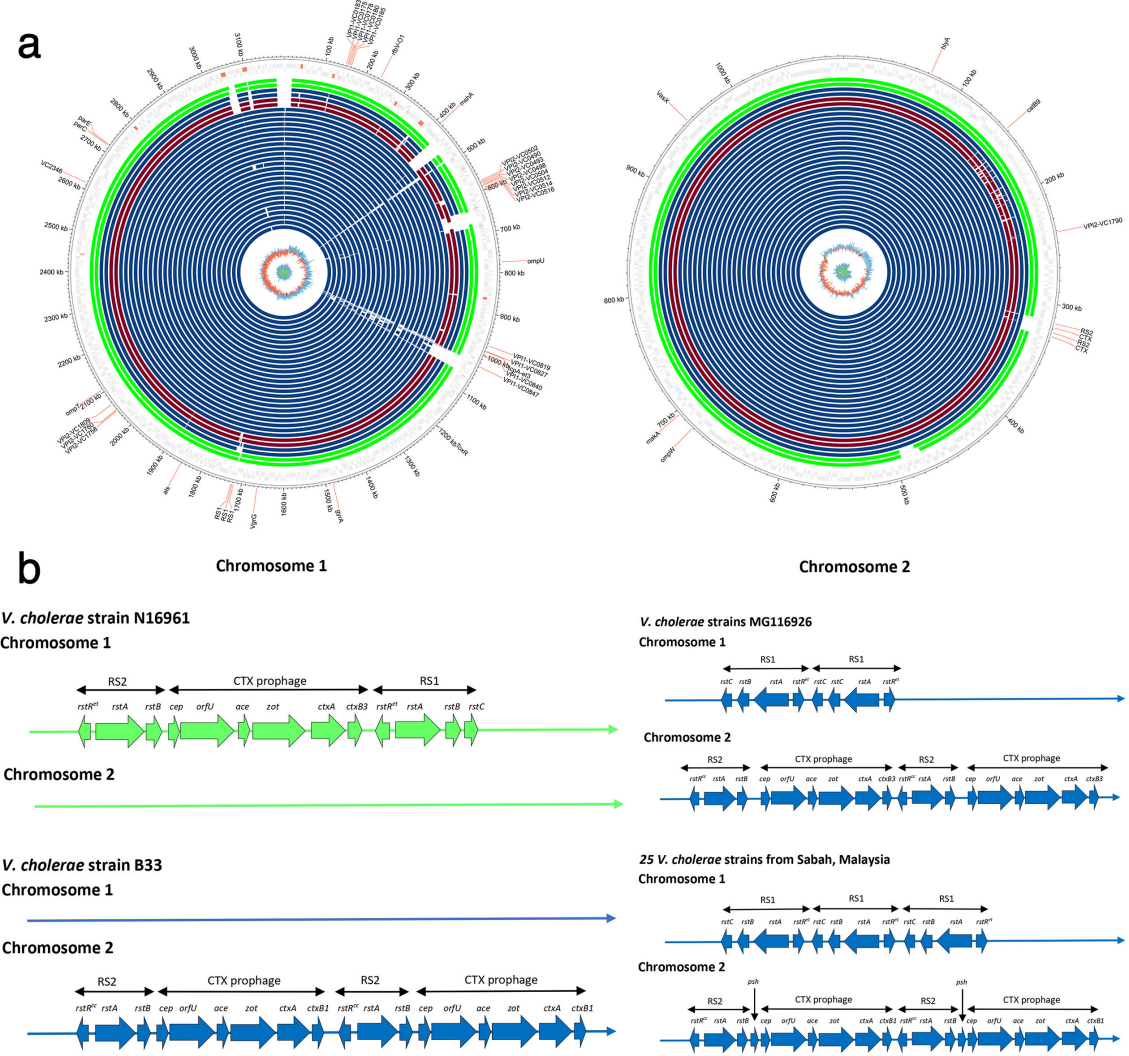
Comparative genomic analysis of pandemic *V. cholerae* strains generated using Circos. The genome visualization highlights key loci, including VPI1, VPI2, biotype-specific genes, and other important genetic regions. (**a**) From the outermost track: reference *V. cholerae* strains N16961 and C5 from wave 1 (green track), reference *V. cholerae* strains B33 and MG116226 from wave 2 (blue track), and reference *V. cholerae* strains 7Mo and S002604 from wave 3 (red track). The subsequent blue tracks represent the 25 *V. cholerae* strains from Sabah, Malaysia, with the complete *V. cholerae* SBHVC14 genome used as a template for the comparison at the center (wave 2). (**b**) Schematic representation of the arrangement of the CTX prophage and RS1 element in *V. cholerae* O1 strains. For comparison, the genetic organization of the CTX prophage, along with the RS1 and RS2 elements, is shown for the El Tor reference strain *V. cholerae* N16961 (AE003852) from wave 1, strain B33 (ACHZ00000000) and the Bangladesh strain MG116926 (GQ485646) from wave 2, alongside the genetic arrangement of the 25 *V. cholerae* strains from Sabah, Malaysia.

**Fig 3:**
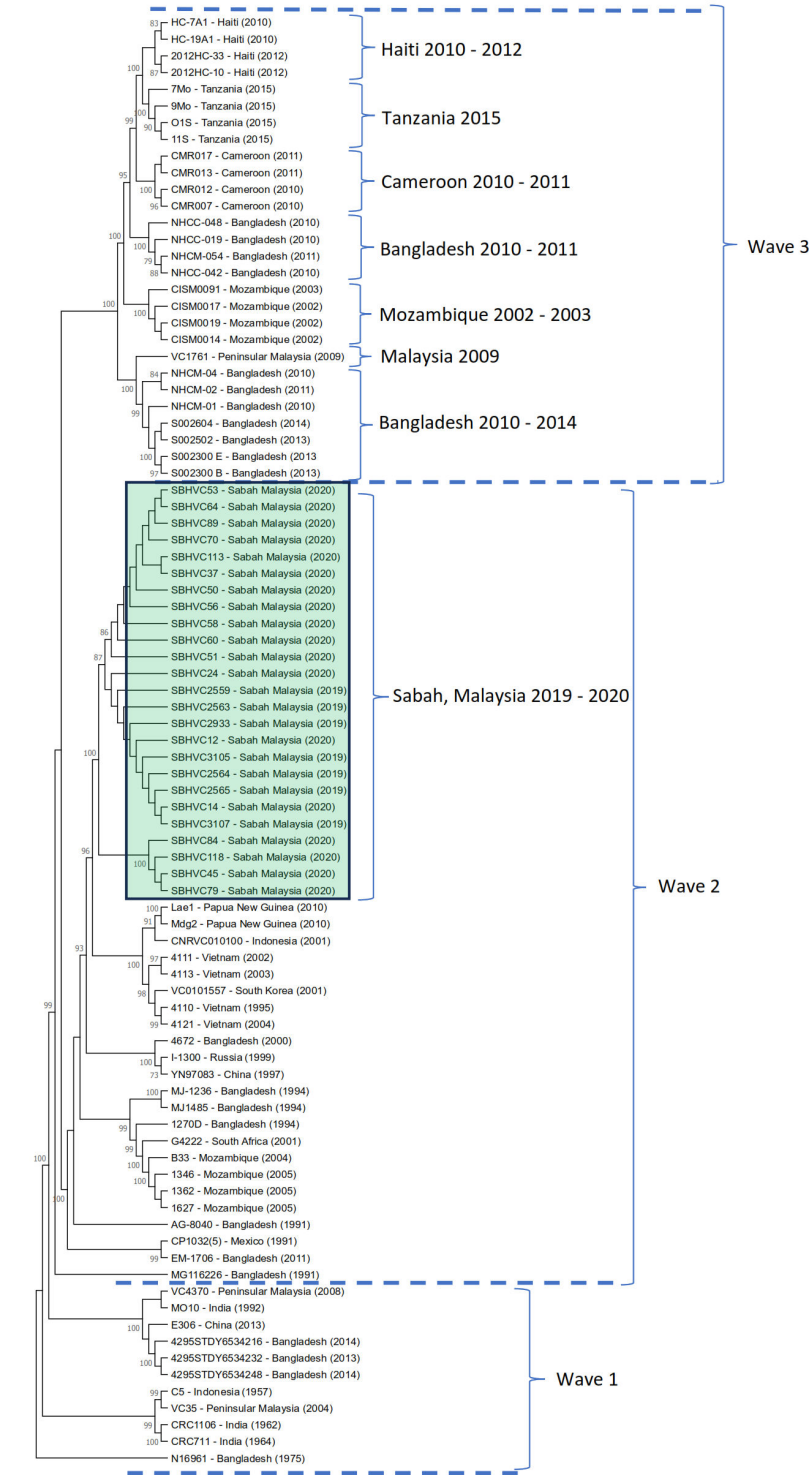
SNP-based phylogenetic tree of 87 seventh-pandemic *V. cholerae* El Tor strains from wave 1, wave 2, and wave 3 outbreaks. Strains from the Sabah 2019–2020 outbreak are highlighted in green. Phylogenetic analysis was performed using the general time reversible (GTR) model with 1,000 bootstrap replicates in the MEGA X software.

A visualization of whole-genome alignment of 24 draft *V. cholerae* genomes and the complete genome of strain SBHVC14 is shown in Fig. 3a. This comparative genomic map offers a circular overview of both chromosomes, providing detailed insights into the genomic architecture and conservation across strains. The alignment reveals a high degree of genome similarity among the strains, with most regions aligning closely to the reference genome. The high degree of genome synteny suggests evolution of all Sabah outbreak strains from a common ancestor, consistent with their phylogeny (Fig. 2). The Sabah strains display all key genetic elements consistent with the seventh pandemic, including the El Tor variant of the toxin coregulated pilus *tcpA* gene, the Vibrio seventh-pandemic islands (VPI1 and VPI2), and the El Tor CTX prophage carrying the cholera toxin *ctxA* and *ctxB* genes (14). This analysis also highlights the presence of VPI1, specifically the *VC2346* gene in Sabah strains (Table S4), which is one of the most acquired genes associated with the seventh-pandemic strain (15). In addition, two other groups of VPI1-associated genes were identified. The first group includes *VC0183*, *VC0175*, *VC017*8, *VC0180*, and *VC0185*, located approximately between 100 and 200 kb on chromosome 1. The second group comprises *VC0819*, *VC0827*, *VC0840*, and *VC0847*, located at around 1,000 kb on chromosome 1.

VPI2 plays an important role in enhancing the bacterium’s ability to survive and thrive within the host (16). It contains genes involved in sialic acid metabolism and neuraminidase production, allowing *V. cholerae* to degrade mucin in the intestinal lining and use sialic acid as a nutrient source (17). VPI2 was identified within the region spanning approximately 550–600 kb on chromosome 1 (*VC0490*, *VC0493*, *VC0498*, *VC0502*, *VC0504*, *VC0512*, *VC0514*, and *VC0516*) and between 2,000 and 2,100 kb (*VC1758*, *VC1760*, and *VC1809*). Additionally, *VC1790* encoding transposase was identified on chromosome 2. All these genetic features, including VPI1, VPI2, CTX prophage, VPI1, VPI2, and RS1, represent horizontally acquired DNA segments that have transformed *V. cholerae* into a highly virulent and human-adapted pathogen (18). These elements form the foundation for the bacterium’s ability to persist in environmental reservoirs and spread effectively during epidemics. The genomic map highlighted the distribution of biotype-specific genes distinguishing Classical and El Tor strains (Fig. 3a; Table S2), supporting their classification and potential lineage-specific adaptations. It also revealed structural variations such as insertions, deletions, and rearrangements, which may involve mobile elements or phage regions contributing to virulence and environmental adaptation. This overview complements phylogenetic analysis and provides a basis for further functional studies.

To further investigate the biotypes of outbreak strains in Sabah, representative biotype-specific genetic markers, including *ctxB1*, *rstR^cc^*, *rstR^et^*, and *tcpA^et^* were analyzed (19, 20). The *rstR^et^* and *tcpA^et^* genes were highly similar to those of the El Tor *V. cholerae* O1 El Tor strain N16961 (AE003852 and AF325734). On chromosome 2, *ctxB1* resembled the sequence found in O1 *V. cholerae* (CP001235), whereas *rstR^cc^* was similar to the sequence from O139 *V. cholerae* (KJ023707). Additionally, RS1 elements were detected in chromosome 1, where three tandem copies of RS1 were arranged as *rstC*, *rstB*, *rstA*, and *rstR^et^* in the studied *V. cholerae* strains. This genetic arrangement is characteristic of the RS1 structure observed in the El Tor variant strains from Bangladesh, which were reported in 2002 (21, 22).

All epidemic and pandemic *V. cholerae* strains are lysogens of the CTX prophage. In Sabah isolates, the 6.5 kb CTX prophage present in chromosome 2 was found to be organized into two repeating units: a 2.4 kb Repeat Sequence 2 (RS2) and a 4.6 kb CTX prophage (Fig. 2b) (21, 22). RS2 contains three genes (*rstR^cc^*, *rstA*, and *rstB*), which are essential for regulation, replication, and integration, respectively. The lysogeny of CTX is primarily regulated by the phage repressor protein *rstR^cc^*, which binds to the *rstA* promoter region and represses the transcription of *rstA* and *rstB*, ultimately affecting CTX production (21, 22).

The observed genetic variability in the CTX prophage and RS1/RS2 elements among *V. cholerae* strains highlights the dynamic nature of its genome architecture. The presence of duplicated CTX prophage regions in the Mozambique *V. cholerae* strain B33, along with strain-specific arrangements of virulence genes (*cep*, *orfU*, *ace*, *zot*, *ctxA*, *ctxB1*, and R2 elements) (23) suggests potential mechanisms for enhanced toxin production or transmission efficiency. Such duplications may arise from recombination events or prophage integration, contributing to the evolutionary success of certain lineages. The high variability in RS1 elements, particularly the repetitive occurrences of *rstC*, *rstB*, and *rstR^et^*, underscores the role of mobile genetic elements in shaping *V. cholerae* diversity (24). These rearrangements could facilitate adaptive advantages, such as antibiotic resistance or immune evasion, by enabling rapid genomic changes. The hybrid architectures observed in some strains, including integrations of RS2 and colonization factors, further support the idea that horizontal gene transfer plays a key role in this pathogen’s evolution.

While this study provides insights into the structural diversity of CTX-related elements, the functional consequences of these genetic variations remain to be fully elucidated (20). Future work should investigate whether specific arrangements correlate with clinical outcomes, environmental persistence, or epidemic potential. Additionally, experimental studies could clarify how these genomic features influence virulence regulation and host-pathogen interactions. In summary, the plasticity of CTX prophage and RS1/RS2 elements in *V. cholerae* reflects its capacity for rapid adaptation. Understanding these genetic dynamics is critical for tracking emerging strains and developing targeted public health interventions.

### Wave 2 El Tor strains are responsible for recent outbreaks in Sabah

To determine the phylogenetic relationship of *V. cholerae* strains from Sabah and put them in the context of the seventh pandemic, we added 62 reference genomes from three major waves of global cholera spread to reconstruct their phylogeny (Fig. 3). These 62 genomes were carefully selected to capture genetic diversity across different geographic regions and time periods, ensuring the inclusion of well-characterized strains previously used in global phylogenetic frameworks. This approach balanced computational efficiency and phylogenetic clarity, allowing the Sabah isolates to be accurately positioned within the established wave 1, wave 2, and wave 3 lineages without introducing redundancy or analytical noise from thousands of highly similar genomes (8, 25, 26). The SNP-based phylogenetic tree revealed distinct clustering patterns corresponding to the known cholera pandemic waves. Strains from Haiti (2010–2012), Cameroon (2010–2011), Bangladesh (2010–2011), Tanzania (2015), and Mozambique (2002–2010) showed close phylogenetic relationships, forming clusters within a larger wave 2 clade. These groupings support earlier studies suggesting transcontinental transmission routes during this period. Surprisingly, despite their more recent isolation, *V. cholerae* strains isolated from the Sabah 2019−2020 outbreak were also found to cluster within wave 2, suggesting that they share a common ancestor with older strains from South Asia and Africa. This finding highlights the ongoing global circulation of wave 2 *V. cholerae*. Furthermore, the monophyly of all Sabah strains suggests a single introduction into the state and subsequent spread. Two subclades were identified among the Sabah isolates, with the major cluster comprising 21 strains that appeared first during the 2019–2020 outbreak, and the minor cluster containing four strains, indicating subsequent genetic diversification following the initial introduction into Sabah.

The study of *V. cholerae* strain MG116926, reported by Nair et al. (21) and Safa et al. (22), shows that strain MG116926 belongs to the Matlab variant III hybrid strain. This strain contains a tandem repeat of the classical CTX prophage on chromosome 2 and a tandem repeat of the RS1 element on the larger chromosome 1, as shown in Fig. 2b. This genetic structure is similar to variants identified in Bangladesh (South Asia) and is classified as a new variant of *V. cholerae* O1. Among the 25 clinical strains, the presence of a tandem repeat of the RS1 element on chromosome I was observed, which is characteristic of the Atypical El Tor biotype of the El Tor lineage (21, 22). The most significant difference compared to the 25 clinical strains is the presence of a tandem repeat of RS1 on chromosome 1, where three copies are present (Fig. 2b). This feature indicates a new variant of *V. cholerae* O1 Biotype El Tor as an Atypical El Tor responsible for the outbreak in Sabah. Another unique characteristic is the presence of the *V2364* marker. Additionally, the 25 isolate strains belonged to the wave 2 *V. cholerae* clades, retaining the *ctxB1* classical cholera toxin B subunit allele while exhibiting an El Tor *tcpA* allele (23, 27). Phylogenetic analysis based on whole-genome SNPs also showed and confirmed that the 25 isolate strains clustered within wave 2 strains.

Genome mapping and SNP-based phylogenetic analysis placed the Sabah strains within the seventh-pandemic lineage, confirming their global pandemic lineage status. Despite their classification, these isolates exhibited sufficient genomic divergence to suggest regional adaptation, supported by the clustering of Sabah isolates in a distinct phylogenetic clade. Genome mapping and SNP-based phylogenetic analysis placed the Sabah strains within the second wave of the seventh-pandemic lineage, confirming their global pandemic lineage status. In addition, the Lae1, Mdg2, and CNRVC010100 strains, which belong to wave 2, were reported to lack the SXT or GI-15 elements, and their antimicrobial resistance genes were not clustered together, as shown in Fig. 3 (26). This finding indicates the absence of genetic relatedness and the lack of SXT, GI-15, or antimicrobial resistance genes among the 25 *V. cholerae* strains isolated from Sabah.

Whole-genome assemblies of 25 *V. cholerae* strains isolated during the 2019–2020 cholera outbreak in Sabah were successfully obtained, revealing comprehensive genomic features across all samples (Table S2). The number of contigs per assembly ranged from 2 to 92 contigs. The total genome lengths were consistent across all strains, ranging between 4,259,716 and 4,293,028 bp. GC content was remarkably consistent among all strains, with each displaying a GC percentage of approximately 45.47%, which is typical for *V. cholerae*. These findings confirm the high quality and consistency of the genome assemblies suitable for downstream comparative and phylogenomic analyses.

### Conclusion

This investigation represents the first complete genomic characterization of *V. cholerae* in Sabah, revealing hybrid biotype traits associated with the second global wave of cholera when compared with isolates from Peninsular Malaysia. Interestingly, wave 2 has been largely replaced by its wave 3 descendants in most parts of the world, making its continued presence and dominance in recent outbreaks in West Malaysia unexpected. This persistence may reflect limited genomic surveillance in Southeast Asia or indicate specific ecological adaptations that confer competitive advantages to wave 2 strains in the coastal environment of Sabah. Such adaptation may reduce the introduction and establishment of newer strains due to local environmental resilience and restricted population movement.

The use of both PacBio and Illumina sequencing platforms proved essential for resolving repetitive and biotype-specific genomic structures, particularly the CTX prophage arrays. The hybrid sequencing strategy enhanced genomic resolution and accuracy, demonstrating its value in molecular epidemiology for tracking pathogen evolution and transmission. As sequencing technologies become more affordable and portable, their integration into regional laboratories will enable faster, evidence-based public health responses.

From a public health perspective, the detection of hybrid and potentially more virulent *V. cholerae* strains underscores the urgent need to strengthen environmental and clinical surveillance systems. Diagnostic protocols should be updated to account for genomic variability, including hybrid biotype markers, to improve detection accuracy. Intervention strategies should prioritize environmental monitoring of coastal reservoirs, rapid molecular diagnostics for outbreak detection, and genomic-based tracking of emerging variants. Furthermore, genomic data can inform vaccine efficacy assessments, guide antimicrobial use policies, and support the development of early warning systems for future cholera outbreaks in Malaysia and neighboring regions.

## Data Availability

The data sets generated and analyzed in this study have been deposited in NCBI GenBank and are publicly available under two BioProjects: PRJNA1270693 and PRJNA1270707. The corresponding 25 genome accession numbers are CP195311–CP195312, JBPPTK000000000, JBPBBT000000000, JBPBBQ000000000, JBPBBS000000000, JBPBBR000000000, JBPBBU000000000, JBPBBV000000000, JBPBBW000000000, JBPBBX000000000, JBPBBY000000000, JBPBBZ000000000, JBPBCA000000000, JBPBCB000000000, JBPBCC000000000, JBPBCD000000000, JBPBCE000000000, JBPBCF000000000, JBPEHP000000000, JBPBBK000000000, JBPBBL000000000, JBPBBM000000000, JBPBBN000000000, JBPBBP000000000, and JBPBBO000000000.

## SUPPLEMENTAL MATERIAL

Tables S1 and S2 spectrum.02191-25-s0001.docx Demographic distribution of *V. cholerae* clinical isolates in Sabah, Malaysia.

Table S3 spectrum.02191-25-s0002.docx Information for the 62 genomes published genomes retrieved from the NCBI database.

Table S4 spectrum.02191-25-s0003.xlsx Details of genes identified using Cholerafinder.

ASM does not own the copyrights to Supplemental Material that may be linked to, or accessed through, an article. The authors have granted ASM a non-exclusive, world-wide license to publish the Supplemental Material files. Please contact the corresponding author directly for reuse.
