# Supplementary material for: Atypical El Tor Vibrio cholerae from the second major global seventh-pandemic cholera wave is endemic in Sabah, Malaysia
Source: Microbiol Spectr. 2026 Feb 9;14(3):e02191-25. doi: 10.1128/spectrum.02191-25 (PMC12955491; doi:10.1128/spectrum.02191-25)
Supplement: Tables S1 and S2 — Demographic distribution of V. cholerae clinical isolates in Sabah, Malaysia. [file spectrum.02191-25-s0001.docx]

**Supplementary Table S1: Demographic Distribution of *V. cholerae* Clinical Isolates in Sabah, Malaysia**

| **Sample Isolate** | **District** | **Gender (Age)** | **Year** |
| --- | --- | --- | --- |
| SBHVC2559 | Putatan | F (40) | 2019 |
| SBHVC2563 | Putatan | F (4) | 2019 |
| SBHVC2564 | Putatan | M (64) | 2019 |
| SBHVC2565 | Putatan | M (12) | 2019 |
| SBHVC2933 | Putatan | M (41) | 2019 |
| SBHVC3105 | Putatan | F (13) | 2019 |
| SBHVC3107 | Putatan | M (4) | 2019 |
| SBHVC12 | Tawau | F (41) | 2020 |
| SBHVC14 | Tawau | F (na) | 2020 |
| SBHVC24 | Kota Kinabalu | M (41) | 2020 |
| SBHVC37 | Kota Kinabalu | F (18) | 2020 |
| SBHVC45 | Kota Kinabalu | F (20) | 2020 |
| SBHVC50 | Putatan | F (19) | 2020 |
| SBHVC51 | Kota Kinabalu | F (24) | 2020 |
| SBHVC53 | Semporna | F (na) | 2020 |
| SBHVC56 | Semporna | M (83 | 2020 |
| SBHVC58 | Federal Territory of Labuan | F (na) | 2020 |
| SBHVC60 | Semporna | F (na) | 2020 |
| SBHVC64 | Semporna | M (43) | 2020 |
| SBHVC70 | Semporna | F (na) | 2020 |
| SBHVC79 | Semporna | F (na) | 2020 |
| SBHVC84 | Semporna | M (na) | 2020 |
| SBHVC89 | Semporna | F (na) | 2020 |
| SBHVC113 | Semporna | F (na) | 2020 |
| SBHVC118 | Semporna | F (na) | 2020 |

**Supplementary Table S2: Assembly Genome Characteristics of *V. cholerae***

| Sample | No. contigs | Total Length (bp) | Longest contig (bp) | N50 (bp) | GC% | Status | Accession number |
| --- | --- | --- | --- | --- | --- | --- | --- |
| SBHVC14 | 2 | 4,293,028 | 3,197,686 | 3,197,686 | 45.46 | *Complete | CP195311 |
|  |  |  |  |  |  |  | CP195312 |
| SBHVC12 | 77 | 4,259716 | 2,003,092 | 590,683 | 45.47 | Draft | JBPPTK000000000 |
| SBHVC24 | 70 | 4,260,338 | 2,003,092 | 590,685 | 45.47 | Draft | JBPBBT000000000 |
| SBHVC37 | 86 | 4,259,946 | 2,003,095 | 590,685 | 45.47 | Draft | JBPBBQ000000000 |
| SBHVC45 | 91 | 4,259,519 | 1,987,265 | 590,684 | 45.47 | Draft | JBPBBS000000000 |
| SBHVC50 | 80 | 4,260,268 | 2,003,095 | 590,683 | 45.47 | Draft | JBPBBR000000000 |
| SBHVC51 | 91 | 4,260,961 | 2,003,095 | 590,685 | 45.47 | Draft | JBPBBU000000000 |
| SBHVC53 | 85 | 4,259,420 | 2,003,095 | 590,684 | 45.47 | Draft | JBPBBV000000000 |
| SBHVC56 | 87 | 4,260,134 | 2,003,074 | 590,685 | 45.47 | Draft | JBPBBW000000000 |
| SBHVC58 | 83 | 4,260,200 | 2,003,085 | 590,685 | 45.47 | Draft | JBPBBX000000000 |
| SBHVC60 | 81 | 4,260,438 | 2,003,096 | 590,685 | 45.47 | Draft | JBPBBY000000000 |
| SBHVC64 | 87 | 4,260,244 | 2,003,095 | 590,685 | 45.47 | Draft | JBPBBZ000000000 |
| SBHVC70 | 85 | 4,259,795 | 2,003,094 | 590,684 | 45.47 | Draft | JBPBCA000000000 |
| SBHVC79 | 80 | 4,260,025 | 1,987,268 | 590,685 | 45.47 | Draft | JBPBCB000000000 |
| SBHVC84 | 91 | 4,260,618 | 1,987,267 | 590,685 | 45.47 | Draft | JBPBCC000000000 |
| SBHVC89 | 90 | 4,260,112 | 2,003,098 | 590,683 | 45.47 | Draft | JBPBCD000000000 |
| SBHVC113 | 87 | 4,259,924 | 2,003,093 | 590,685 | 45.47 | Draft | JBPBCE000000000 |
| SBHVC118 | 92 | 4,260,252 | 1,987,269 | 590,683 | 45.47 | Draft | JBPBCF000000000 |
| SBHVC2559 | 65 | 4,260,618 | 2,004,092 | 597,285 | 45.47 | Draft | JBPEHP000000000 |
| SBHVC2563 | 67 | 4,260,831 | 2,003,095 | 590,679 | 45.47 | Draft | JBPBBK000000000 |
| SBHVC2564 | 66 | 4,261,159 | 2,003,093 | 590,683 | 45.47 | Draft | JBPBBL000000000 |
| SBHVC2565 | 67 | 4,260,469 | 2,003,186 | 590,685 | 45.47 | Draft | JBPBBM000000000 |
| SBHVC2933 | 63 | 4,260,473 | 2,003,096 | 590,678 | 45.47 | Draft | JBPBBN000000000 |
| SBHVC3105 | 58 | 4,263,193 | 2,003,102 | 590,685 | 45.47 | Draft | JBPBBP000000000 |
| SBHVC3107 | 65 | 4,261,525 | 2,004,182 | 590,683 | 45.47 | Draft | JBPBBO000000000 |
