# Supplementary material for: Atypical El Tor Vibrio cholerae from the second major global seventh-pandemic cholera wave is endemic in Sabah, Malaysia
Source: Microbiol Spectr. 2026 Feb 9;14(3):e02191-25. doi: 10.1128/spectrum.02191-25 (PMC12955491; doi:10.1128/spectrum.02191-25)
Supplement: Table S3 — Information for the 62 genomes published genomes retrieved from the NCBI database. [file spectrum.02191-25-s0002.docx]

**Supplementary Table S3:** Information for the 62 genomes published genomes retrieved from the NCBI database.

| Strain Name | Year | Country | Num. of Contigs |  | Genome Size | Wave | Accession |
| --- | --- | --- | --- | --- | --- | --- | --- |
| MO10 | 1992 | India | 27 |  | 4,079,638 | 1 | GCA_000152425.1 |
| N16961 | 1975 | Bangladesh | 2 |  | 4,033,464 | 1 | GCA_000006745.1 |
| E306 | 2013 | China | 51 |  | 4,165,066 | 1 | GCA_000487955.1 |
| C5 | 1957 | Indonesia | 2 |  | 4,102,038 | 1 | GCA_001887395.1 |
| CRC711 | 1964 | India | 2 |  | 4,057,520 | 1 | GCA_001887435.1 |
| CRC1106 | 1962 | India | 2 |  | 4,099,119 | 1 | GCA_001887455.1 |
| 4295STDY6534232 | ND | ND | 2 |  | 4,092,645 | 1 | GCA_900324425.1 |
| 4295STDY6534216 | ND | ND | 2 |  | 4,092,641 | 1 | GCA_900324445.1 |
| 4295STDY6534248 | ND | ND | 2 |  | 4,092,644 | 1 | GCA_900324455.1 |
| G4222 | 2001 | South Africa | 2 |  | 4,202,811 | 2 | GCA_000338075.1 |
| I-1300 | 1999 | Russia | 2 |  | 4,033,785 | 2 | GCA_000967785.1 |
| CP1032(5) | 1991 | Mexico | 17 |  | 3,971,467 | 2 | GCA_000279305.1 |
| YN97083 | 1997 | China | 64 |  | 4,074,242 | 2 | GCA_001030015.1 |
| B33 | 2004 | Mozambique | 17 |  | 4,154,698 | 2 | GCA_000174315.1 |
| 4121 | 2004 | Vietnam | 47 |  | 3,948,118 | 2 | GCA_001252075.1 |
| 4110 | 1995 | Vietnam | 97 |  | 3,918,727 | 2 | GCA_001257035.1 |
| 4113 | 2003 | Vietnam | 39 |  | 3,925,537 | 2 | GCA_001259055.1 |
| 1362 | 2005 | Mozambique | 40 |  | 4,064,557 | 2 | GCA_001260295.1 |
| EM-1706 | 2011 | Bangladesh | 88 |  | 3,944,903 | 2 | GCA_001186645.1 |
| 4672 | 2000 | Bangladesh | 199 |  | 3,972,315 | 2 | GCA_001249795.1 |
| MJ-1236 | 1994 | Bangladesh | 2 |  | 4,236,368 | 2 | GCA_000022585.1 |
| MJ1485 | 1994 | Bangladesh | 68 |  | 4,112,661 | 2 | GCA_001250195.1 |
| 4111 | 2002 | Vietnam | 132 |  | 3,870,008 | 2 | GCA_001252855.1 |
| 1346 | 2005 | Mozambique | 40 |  | 4,069,009 | 2 | GCA_001253035.1 |
| MG116226 | 1991 | Bangladesh | 46 |  | 4,028,960 | 2 | GCA_001254355.1 |
| 1627 | 2005 | Mozambique | 36 |  | 4,065,829 | 2 | GCA_001247835.1 |
| AG-8040 | 1991 | Bangladesh | 103 |  | 3,977,194 | 2 | GCA_000348145.2 |
| VC0101557 | 2001 | South Korea | 54 |  | 3,863,050 | 2 | GCA_002407455.1 |
| 1270D | 1994 | Russia | 133 |  | 4,016,316 | 2 | GCA_003130495.1 |
| HC-70A1 | ND | Haiti | 169 |  | 4,057,434 | 3 | GCA_000221385.1 |
| HC-19A1 | 2010 | Haiti | 96 |  | 4,033,401 | 3 | GCA_000234965.1 |
| 2012HC-33 | 2012 | Haiti | 107 |  | 4,014,752 | 3 | GCA_000788975.1 |
| 2012HC-10 | 2012 | Haiti | 108 |  | 4,012,032 | 3 | GCA_000789135.1 |
| NHCC-048 | 2010 | Bangladesh | 113 |  | 4,041,973 | 3 | GCA_001186675.1 |
| NHCC-042 | 2010 | Bangladesh | 101 |  | 4,040,251 | 3 | GCA_001186735.1 |
| NHCC-019 | 2010 | Bangladesh | 249 |  | 4,006,456 | 3 | GCA_001186755.1 |
| NHCM-01 | 2010 | Bangladesh | 129 |  | 4,058,157 | 3 | GCA_001186825.1 |
| NHCM-02 | 2011 | Bangladesh | 89 |  | 4,058,043 | 3 | GCA_001186855.1 |
| NHCM-04 | 2011 | Bangladesh | 87 |  | 4,051,907 | 3 | GCA_001186905.1 |
| NHCM-054 | 2011 | Bangladesh | 79 |  | 4,037,409 | 3 | GCA_001187165.1 |
| CMR007 | 2010 | Cameroon | 261 |  | 4,026,765 | 3 | GCA_001860265.1 |
| CMR012 | 2011 | Cameroon | 199 |  | 4,027,803 | 3 | GCA_001860365.1 |
| CMR013 | 2011 | Cameroon | 161 |  | 4,034,667 | 3 | GCA_001860385.1 |
| CMR017 | 2011 | Cameroon | 149 |  | 4,034,135 | 3 | GCA_001860465.1 |
| CISM_0091 | 2003 | Mozambique | 95 |  | 4,036,548 | 3 | GCA_002098885.1 |
| CISM_0019 | 2002 | Mozambique | 109 |  | 4,033,489 | 3 | GCA_002098965.1 |
| CISM_0017 | 2002 | Mozambique | 107 |  | 4,036,556 | 3 | GCA_002099015.1 |
| CISM_0014 | 2002 | Mozambique | 118 |  | 4,032,716 | 3 | GCA_002099065.1 |
| O1S | 2015 | Tanzania | 101 |  | 4,024,114 | 3 | GCA_002076155.1 |
| 11S | 2015 | Tanzania | 99 |  | 4,025,935 | 3 | GCA_002076185.1 |
| 7Mo | 2015 | Tanzania | 95 |  | 4,022,979 | 3 | GCA_002076615.1 |
| 9Mo | 2015 | Tanzania | 102 |  | 4,024,326 | 3 | GCA_002076635.1 |
| S002502 | 2013 | Bangladesh | 77 |  | 3,946,275 | 3 | GCA_002807725.1 |
| S002300_E | 2013 | Bangladesh | 77 |  | 4,042,300 | 3 | GCA_002807985.1 |
| S002300_B | 2013 | Bangladesh | 75 |  | 3,949,769 | 3 | GCA_002807835.1 |
| S002604 | 2014 | Bangladesh | 78 |  | 4,045,052 | 3 | GCA_002808155.1 |
| VC1761 | 2009 | Malaysia | 149 |  | 4,011,457 | 3 | GCA_000299515.2 |
| VC4370 | 2008 | Malaysia | 145 |  | 3,986,677 | 1 | GCA_000299535.2 |
| VC35 | 2004 | Malaysia | 103 |  | 3,912,714 | 1 | GCA_000299495.1 |
| Lae1 | 2010 | Papua New Guinea | 67 |  | 4,000,180 | 2 | GCA_027801255.1 |
| Mdg2 | 2010 | Papua New Guinea | 69 |  | 3,968,294 | 2 | GCA_027801275.1 |
| CNRVC010100 | 2001 | Indonesia | - |  | - | 2 | ERR1877943 |
